# A Narrative Review of Current Striae Treatments

**DOI:** 10.3390/healthcare10122565

**Published:** 2022-12-17

**Authors:** Nuno Mendes, Paulo Jorge Alves, Mafalda Barros, Jorge Magalhães Rodrigues, Jorge Machado

**Affiliations:** 1 ICBAS—School of Medicine and Biomedical Sciences, Porto University, 4050-313 Porto, Portugal; 2 CNM—Clínica Nuno Mendes do Grupo Saúde Nuno Mendes, 4560-164 Penafiel, Portugal; 3 Centro Regional do Porto, ICS—Instituto de Ciências da Saúde da Universidade Católica Portuguesa, 4169-005 Porto, Portugal; 4 CBSin—Center of BioSciences in Integrative Health, 4250-105 Porto, Portugal

**Keywords:** stretch marks, striae, quality of life, treatment, prevention

## Abstract

Striae (*striae cutis distensae*) are a common disfiguring skin condition, characterised by the appearance of linear bands on the skin, with an atrophic look. The striae development is still unknown, being more common in women than in men. The prevalence of this condition ranges from 50% to 90%. Regarding treatment, there are various treatment strategies currently available for stretch marks, including topical preparations such as tretinoin and glycolic acid, and also laser. The goal of this work is to discover the main treatments available for striae management. For that, a search was performed based on the definition of specific scientific keywords, by exploring PubMed, ScienceDirect and Biblioteca Virtual em Saúde (BVS). MeSH thesaurus (Medical Subject Headings) descriptors were used. The results indicate that, to date, no treatment is fully effective. More clinical trials are needed to validate the efficacy of these therapies and their long-term use in this type of skin lesion.

## 1. Introduction

*Striae* (*striae cutis distensae*), known as stretch marks, are a common disfiguring skin condition, characterised by the appearance of atrophic-looking linear bands in the skin [1,2,3]. The cause of this pathology is currently unknown; however, stretch marks can develop in numerous physiological and pathological states, such as in pregnancy, adolescent growth, obesity, large weight gain, patients with Cushing syndrome and Marfan syndrome, diabetes mellitus, and in situations of long-term use of systemic and topical steroids [1,2].

*Striae* are classified according to their appearance or epidemiology [2], such as:

- Atrophic stretch marks (tapered skin);
- *Distensae* stretch marks (stretched skin);
- Black stretch marks;
- *Striae rubrae* (red)/*Striae albae* (white);
- *Striae gravidarum* (in pregnancy);
- Striae caerulea (dark blue).

Clinically, newly formed *striae* appear initially as linear pink or purple lesions, without substantial skin depression (*striae rubra*) [1,3]. Over time, this lesion tends to lose its pigmentation, becoming atrophic and white (*striae albae*). The most frequently affected body sites are the breasts, forearms, abdomen, buttocks, and thighs [2].

Regarding the progression of *striae*, although unknown, it is thought that involves macrophage activity and the release of elastases from mast cells [2]. On one hand, *striae rubrae* presents an excess of thin elastic fibres in the papillary dermis area, with the presence of thicker fibres in the periphery, with vasodilation and oedema. There is also a decrease in the elastin and fibrillin fibres and structural changes in collagen fibres [2]. On the other hand, *striae albae* reveal epidermal atrophy, less vascularisation, and more dense, thin, and cicatrised horizontal collagen lines [1,2,3]. Several recent electron microscopy studies have shown indications of mast cell degranulation, macrophage activation, and dermal elastolysis [2].

Stretch marks are more common in women than in men, being more prominent in dark-skinned individuals [1,2]. In puberty, the *striae* prevalence ranges from 6% to 86%, and, in obesity, it is 43% [2]. There is also a higher prevalence in individuals with a larger abdominal area and weight gain [4]. During pregnancy, where stretch marks are known as *striae gravidarum*, they range between 50% and 90% [4], being more common in younger women. A Japanese study demonstrated that stretch marks affected the quality of life of pregnant women [5]. Even using preventive measures such as a moisturizer, which increased the amount of water in the most extern layer of the epidermis, did not prevent the appearance of stretch marks [5]. Another one showed that multiparous women are specifically affected by this pathology [6]. 

In a study in which skin biopsies were collected from people with and without stretch marks, histochemical assays were performed, assessing total protein, DNA, and elastin, to analyse the stretch marks [7]. The authors evaluated cell migration and the proliferation of primary cell culture from biopsied fibroblasts [7]. These techniques could be used as diagnostic tools to predict the predisposition of stretch marks in the future. The reversibility of fibroblast phenotypes may also be a new perspective of preventing treatment for people predisposed to develop *striae*.

Several authors use various methods to evaluate the type and severity of *striae*, by analysing the treatment’s efficacy. However, they are not standardised or validated. A new BODY-Q scale has been developed and field-tested to measure the appearance of stretch marks, to provide ways to incorporate patients’ perspectives into future treatment studies [8]. This could be used to measure the impact of innovative treatment for *striae*. 

Regarding treatment, there are currently a wide variety of strategies including laser and topical preparations such as tretinoin and glycolic acid [1,3,9]. Hyaluronic acid is also used, but with weak evidence that it prevents stretch marks. Tretinoin is limited in pregnancy [10]. However, the available topic methods generally do not have evidence from rigorous and well-designed controlled trials [10].

To the best of our knowledge, research on this topic is still limited. For this reason, this research intends to make the available scientific evidence known by reflecting the main results on this subject in a clear and summarised way. For that, a review was conducted on studies and articles published in the past 10 years on the subject. Scientific articles were used to select the most important data and thus obtain conclusions that provide a high level of evidence. This study will provide a wide and more concrete view of this issue.

Considering all the above, the main goals are (1) to know the clinical and epidemiologic characteristics of stretch marks, (2) to analyse the physiopathological mechanisms related to *striae* development, (3) to identify the main pathologies related to the *striae* development, and (4) to analyse the efficacy of the currently available treatments for stretch marks and their impact on patients’ quality of life.

## 2. Methods

To assess the current state of the work, a search was conducted in the selected databases: PubMed, ScienceDirect, and Biblioteca Virtual em Saúde (BVS).

Search equations were used, being formulated from the definition of keywords translated into scientific language. We also used the MeSH thesaurus (Medical Subject Headings), which belongs to PubMed, and DeCS (Health Science Descriptors). The keywords used were stretch marks, *striae*, quality of life, treatment, and prevention. To combine these terms, the Boolean or logic operators AND/OR were used.

Only publications in English, Portuguese, and Spanish, from 2010 to March 2020, were considered. 

The CASPE critical reading model was used. This is a program developed by the Oxford Institute of Health Sciences to help health research services to improve their ability to search for information and critical reading skills in the scientific literature [11].

## 3. Discussion

*Striae distensae* are a common dermal lesion that produces significant physical and psychological impact [9], being an anxiety source for women and affecting their quality of life [12]. Although stretch marks do not affect body functions, they are considered disfiguring, since they produce permanent changes in the appearance of the skin, like a scar [1]. They often represent a cause of aesthetic morbidity, especially in women and in certain jobs where physical appearances are of great importance [13]. 

Physiologically, stretch marks are characterised by a marked rupture of elastic fibres, complex structures responsible for the ability to resist forces, that return to their original anatomical position [14]. There is also the appearance of thin and disorganised fibrils that are subsequently synthesised [14]. This rupture in the fibrils leads to changes in the viscoelastic properties in the affected area of the skin, which becomes less firm and less elastic than normal skin [15]. The abdomen, breasts, and thighs are the most affected body areas [13]. The exact cause for the appearance of stretch marks is still unclear, and, so far, there is no effective preparation to prevent their development.

Stretch marks are caused by rapid tissue expansion, a process that can occur during pregnancy, puberty, obesity, and in diseases whose treatment requires the use of corticosteroids [16]. The most common risk factors for developing stretch marks include young age, skin phototype, maternal and family history of stretch marks, higher pre-pregnancy, and pre-labour weight [14]. Knowledge of these risk factors can provide effective support and counselling to each person, especially among younger women, to prevent the development of stretch marks during pregnancy [17]. Several studies already conducted have shown a statistically significant association between these risk factors and the development of *striae gravidarum* (SG) [14]. Another research conducted on 299 Caucasian women showed that although breast *striae* were associated with an increased risk of developing SG, women present a lower risk of developing it on their thighs [18]. In addition to these factors, high alcohol intake and reduced water intake were also found more frequently in women who developed SG [18]. High glucose levels and diabetes may be related to increased development of SG; however, there is no link between these two situations [18].

Regarding epidemiology, the prevalence of stretch marks is variable. It is more frequent in women between 5 and 50 years old [13]. In the specific case of SG, the *striae* prevalence in women is between 50% and 90% [12]. Picard et al. revealed significant differences in the development of SG at younger ages (with an odds ratio of 28.25 for women of 20 years or younger) [17]. For this reason, there is the concept that pregnancy in adolescence is associated with the risk of developing stretch marks. In fact, pregnant adolescents may not only be at higher risk of developing stretch marks, but also of perinatal morbidity and mortality [14]. Regarding SG physiology, the elastic fibrils of these stretch marks are rich in tropoelastin, with the atrophied flattened lesions being also associated with a loss of rete crystals and an increase in glycosaminoglycans [18]. However, the severity and development of the lesions vary between individuals, indicating a variable genetic predisposition [18].

Clinically, *striae* appear as red/purple lines or stripes aligned perpendicular to the direction of skin tension, becoming paler over time [12]. *Striae rubrae* and *albae* can be differentiated by their clinical appearance [15]. Initially, *striae* present erythematous lesions (*rubrae*), but, in the chronic phase, they develop into hypopigmented atrophic scars (*albae*) [16]. Kim, M., Kim, S., and Jung, Y., et al. decided to evaluate the biophysical properties of *striae distensae*, *rubrae*, and *albae* to understand the etiological mechanism and evaluate the efficacy of treatments, considering skin surface structure, skin lightness, and hydration, and found significant differences in the colour of the skin and in various surface structures compared to adjacent normal skin [16]. Regarding skin surface patches, *striae* were more anisotropic and directional, with a more irregular polygonal pattern of each segmented unit compared to the normal skin [16]. Regarding the results of *striae albae,* they showed a wrinkled and atrophied structure, deeper and more voluminous than normal skin [16]. In addition, while the *striae albae* showed more brightness and less redness, the *striae rubra* did not [16].

Currently, there are several therapeutic modalities available for the treatment and prevention of stretch marks, but none of them are completely effective in eradicating this cutaneous pathology, without good scientific evidence [13].

The most used treatment is based on the application of topical products, used therapeutically and prophylactically, such as tretinoin, ascorbic acid, and hyaluronic acid [13]. Hyaluronic acid is considered to stimulate the activity of fibroblasts [9], important cells responsible for maintaining the structure and tissues [12]. Several moisturizers, such as Verum and Alphastria, which combine hyaluronic acid with various vitamins and fatty acids, have been shown to significantly reduce the incidence of SG [18]. However, these moisturizers were applied through massage, which leads to the question if the cream is beneficial, or if it is just a result of the massage [18]. The use of 0.05% tretinoin demonstrates improvements of up to 47% in *striae* patients [19].

In addition to these constituents, topical preparations also contain multiple others, namely emollients, such as isopropyl palmitate, PPG-15 stearic ether (1-octadecoxypropan-2-ol), collagen, hydrolysed elastin, and vitamins A and E [12]. Curiously, Centella Asiatica is an active constituent that stimulates fibroblasts and cell proliferation and is present in some moisturizing creams, such as Trofolastin and Liforma Stretch Mark Gel Day and Night Cream (Liforma, Reigate, U.K.), which prevent the appearance of *striae distensae* [9]. The use of Trofolastin in a controlled trial, when compared with a placebo, showed that, of the 80 subjects who participated, 56% of the placebo group developed *striae distensae*, compared with 34% of the moisturizing cream treatment group [20]. Furthermore, women who had a history of *striae distensae* at puberty stated that Trofolastin treatment was 100% effective in preventing it [20].

For atrophic scar cases resulting from *striae distensae*, the use of a silicone gel is recommended. In a controlled blind trial, the effects of this gel were compared with a placebo, using non-invasive measures and immunohistochemical analysis of tissue biopsies [21]. Both histological evidence and clinical data showed that melanin values increased, while collagen, elastin, and vascularisation values decreased over a six-week period [21]. A product containing *Centella Asiatica* extract demonstrated significant improvements in skin elasticity and a 60% reduction in the existence of stretch marks [9]. Several other creams of this kind have been tested and showed acceptable results, although studies on them have not yet been published. That is the case of a cream that contains shea butter and cocoa butter as active ingredients, and strengthens the skin by increasing collagen levels and reducing the appearance of *striae rubra* and *striae albae* [9]. Other products also promote collagen production [9], treating stretch marks.

Brennan, Young, and Devane discuss six clinical trials involving a total of 800 women, with no statistically significant differences in the development of stretch marks in those who received topical preparations with active ingredients compared to women who received no treatment or received a placebo, or compared to the use of other topical preparations [12]. Another similar study by Ud-Din, McGeorge, and Bayat reports that there are only a few studies (*n* = 11) investigating the efficacy of topical preparations in managing stretch marks [9]. Overall, there is a lack of evidence for each topical formulation.

There is also the use of oils for preventing *striae distensae*, such as almond oil and cocoa butter cream, which prevent and reduce the appearance of stretch marks by maintaining the elasticity of the epidermis and rehydrating the skin [9].

The use of lasers, including carbon dioxide (CO_2_), Er: YAG, diode, Nd: Q-switched YAG, and pulsed and excimer lasers are also increasingly used [13]. Laser therapy represents a considerable advance in the treatment of stretch marks, especially in *striae rubra*, since, as there is an increase in vascularisation of the lesions, vascular lasers have beneficial effects, since haemoglobin functions as a chromophore for this type of laser [15]. In this type of treatment, cooling before and right after the use of the laser is crucial to protect the epidermis, but not too long to avoid vasoconstriction [15]. The use of non-ablative fractional lasers demonstrates improvements of 50$ to 75% in *striae distensae* lesions [18]. The pulsed laser of specifically 585 nanometres (nm) with a fluence of 3.0 J/cm^2^ has been shown to improve the appearance of stretch marks [22] and had a more moderate effect on *striae rubra*, but no beneficial effect on *striae albae* [23]. It is important to note that the use of this technique on ethnic type 4–6 skin should be avoided, due to the risk of permanent pigmentary changes [24]. In addition, the 511–577 nanometre (nm) copper bromide laser was also evaluated in a 2-year pilot study for women with skin phototype type II–III on the Fitzpatrick scale, with beneficial clinical and histological effects on stretch marks [25]. The fractional CO_2_ laser is also shown to be effective on *striae albae*, in subjects with skin types III and IV, being significantly better than topical treatment with 0.05% tretinoin cream and 10% glycolic acid [26], which proves to be the first option to choose in *striae albae* treatment. Results with Er: YAG laser, on the other hand, are limited and the appearance of redness as a consequence tends to last for a long time [15].

Other devices such as radiofrequency, chemical peeling, puncture, carboxytherapy, phototherapy, and therapies such as platelet-rich plasma have also been used with success [13]. Regarding chemical peeling, it is known that applying chemical agents leads to inflammatory responses with subsequent neocalagenosis and mild skin irritation [13]. The most commonly used agents for this process are usually trichloroacetic acid, retinoic acid, and glycolic acid [19,27]. However, applying this peeling can be a cost-effective option for treating *striae distensae* [13]. Carboxytherapy stimulates blood circulation, increasing the release of oxygen [13], which activates the synthesis of collagenase, elastin, and hyaluronic acid, and has been shown to decrease the size of the *striae* [13]. However, it is considered painful and uncomfortable, and thus is a controversial therapy.

Treatments based on high-intensity light emission have moderate efficacy. A study using a high-intensity light device emitting UVB and UVA1 with peak wavelengths at 313, 360, and 420 nm reported improvements of more than 51% in stretch mark pigmentation after 10-week phototherapy sessions, although cases of transient hyperpigmentation were reported [28]. Intense pulsed light (IPL) appears to lead to moderate improvements in stretch marks; however, the presence of erythema and post-inflammatory hyperpigmentation complicate this treatment [29].

Bipolar radiofrequency also shows clinical and histological improvements in *striae distensae*, while tripolar radiofrequency resulted in a 25–75% improvement in just one week of treatment [30]. Needle therapy also shows significant improvements in stretch marks [31] compared with microdermabrasion, although the latter is effective for *striae rubra* [32].

Overall, there are only a limited number of studies conducted that investigate the effectiveness of different types of treatment for stretch marks. More clinical trials will be needed to test more products and techniques for more effective strategies to prevent and treat this condition. We also suggest that future reviews should focus on specific groups of techniques and explore the mechanisms through which they act.

## 4. Conclusions

Stretch marks are a common dermal lesion, with a prevalence between 50% and 90%, generally occurring between the ages of 5 and 50. It affects mainly women during pregnancy, puberty, obesity, and in pathologies whose treatment requires the use of corticosteroids. Functionally, stretch marks do not cause any changes, but they can have a significant physical and psychological impact, affecting the quality of life. They are characterised by changes in the elastic fibre network, leading to the appearance of thin, disorganised, tropoelastin-rich fibres. However, the fundamental biological factor underlying this remodelling remains unknown.

To date, no fully effective treatment has emerged. The most common therapy is the application of topicals such as tretinoin, glycolic acid, ascorbic acid, hyaluronic acid, and *Centella Asiatica*. Other commonly cited treatments include laser therapy (CO_2_, Er: YAG, diode, Nd: Q-switched YAG). In addition to these, radiofrequency, phototherapy, and therapies such as chemical peeling, carboxytherapy, and micro-dermabrasion have been used with varying success.
